# Regional knockdown of NDUFS4 implicates a thalamocortical circuit mediating anesthetic sensitivity

**DOI:** 10.1371/journal.pone.0188087

**Published:** 2017-11-14

**Authors:** Renjini Ramadasan-Nair, Jessica Hui, Pavel I. Zimin, Leslie S. Itsara, Philip G. Morgan, Margaret M. Sedensky

**Affiliations:** 1 Center for Integrative Brain Research, Seattle Children’s Research Institute, 1900 Ninth Avenue, Seattle, WA, United States of America; 2 Department of Anesthesiology and Pain Medicine, University of Washington, Seattle, WA, United States of America; University of Nebraska Medical Center, UNITED STATES

## Abstract

Knockout of the mitochondrial complex I protein, NDUFS4, profoundly increases sensitivity of mice to volatile anesthetics. In mice carrying an *Ndufs4*^lox/lox^ gene, adeno-associated virus expressing Cre recombinase was injected into regions of the brain postulated to affect sensitivity to volatile anesthetics. These injections generated otherwise phenotypically wild type mice with region-specific, postnatal inactivation of *Ndufs4*, minimizing developmental effects of gene loss. Sensitivities to the volatile anesthetics isoflurane and halothane were measured using loss of righting reflex (LORR) and movement in response to tail clamp (TC) as endpoints. Knockdown (KD) of *Ndufs4* in the vestibular nucleus produced resistance to both anesthetics for movement in response to TC. *Ndufs4* loss in the central and dorsal medial thalami and in the parietal association cortex increased anesthetic sensitivity to both TC and LORR. Knockdown of *Ndufs4* only in the parietal association cortex produced striking hypersensitivity for both endpoints, and accounted for half the total change seen in the global KO (*Ndufs4(KO)*). Excitatory synaptic transmission in the parietal association cortex in slices from *Ndufs4(KO)* animals was hypersensitive to isoflurane compared to control slices. We identified a direct neural circuit between the parietal association cortex and the central thalamus, consistent with a model in which isoflurane sensitivity is mediated by a thalamic signal relayed through excitatory synapses to the parietal association cortex. We postulate that the thalamocortical circuit is crucial for maintenance of consciousness and is disrupted by the inhibitory effects of isoflurane/halothane on mitochondria.

## Introduction

Anesthesia is a complex phenomenon characterized by amnesia, analgesia, immobility and loss of consciousness. Anesthetic mechanisms are not well understood at several levels of neuronal function. These include the molecular binding targets, the disrupted physiological mechanisms, the brain regions involved, and the altered neuronal circuitry systems. Previous reports have shown that defects in mitochondrial complex I profoundly hypersensitize *C*. *elegans*, mice and children to volatile anesthetics [1–3]. In addition, complex I function is uniquely sensitive to volatile anesthetics in concentrations that correlate with the whole animal EC_50_s of the respective species [1, 4, 5].

Mice featuring a global knock out of the mitochondrial protein NDUFS4 display biochemically proven complex I specific mitochondrial dysfunction [6–8]. This animal, *Ndufs4(KO)*, displays the greatest volatile anesthetic hypersensitivity yet reported for any mammal [3]. The sensitivity is recapitulated by glutamate-cell specific KO of the gene in the central nervous system [9]. It is well documented that anesthetics lower the cerebral metabolic rate in an agent- and region-specific manner [10–14]. However, whether this is a cause or effect of the anesthetized state is controversial [15, 16]. Changes in anesthetic sensitivity seen in *Ndufs4(KO)* mice favor the model that a lowered metabolic rate causes the anesthetic state. However, it remains possible that compensatory developmental changes in the constitutive global *Ndufs4(KO)* may also play a role in determining anesthetic sensitivity.

To determine whether the inhibited metabolic state of specific regions of the CNS causes the anesthetic phenotype, and to eliminate early developmental changes resulting from the global loss of *Ndufs4*, we postnatally knocked down *Ndufs4* in specific CNS regions in mice with normal volatile anesthetic sensitivity. Taking cues from current literature [17–19] and the pathogenesis of the global KO [20], *Ndufs4* was separately knocked down in the vestibular nucleus (VN), the mesopontine tegmental area (MPTA), central and dorsal medial thalami (CMT & DMT), and the parietal association cortex (PAC). The contribution of the regional mitochondrial defects on sensitivity to isoflurane (ISO) or halothane (HAL) were evaluated using loss of righting reflex (LORR) and loss of response to a tail clamp (TC) as endpoints. Since loss of the protein NDUFS4 in the PAC caused the largest change in ISO and HAL sensitivity, we investigated the effect of NDUFS4 loss on the sensitivity of excitatory field potentials to ISO in the PAC.

## Results

**Fig 1:**
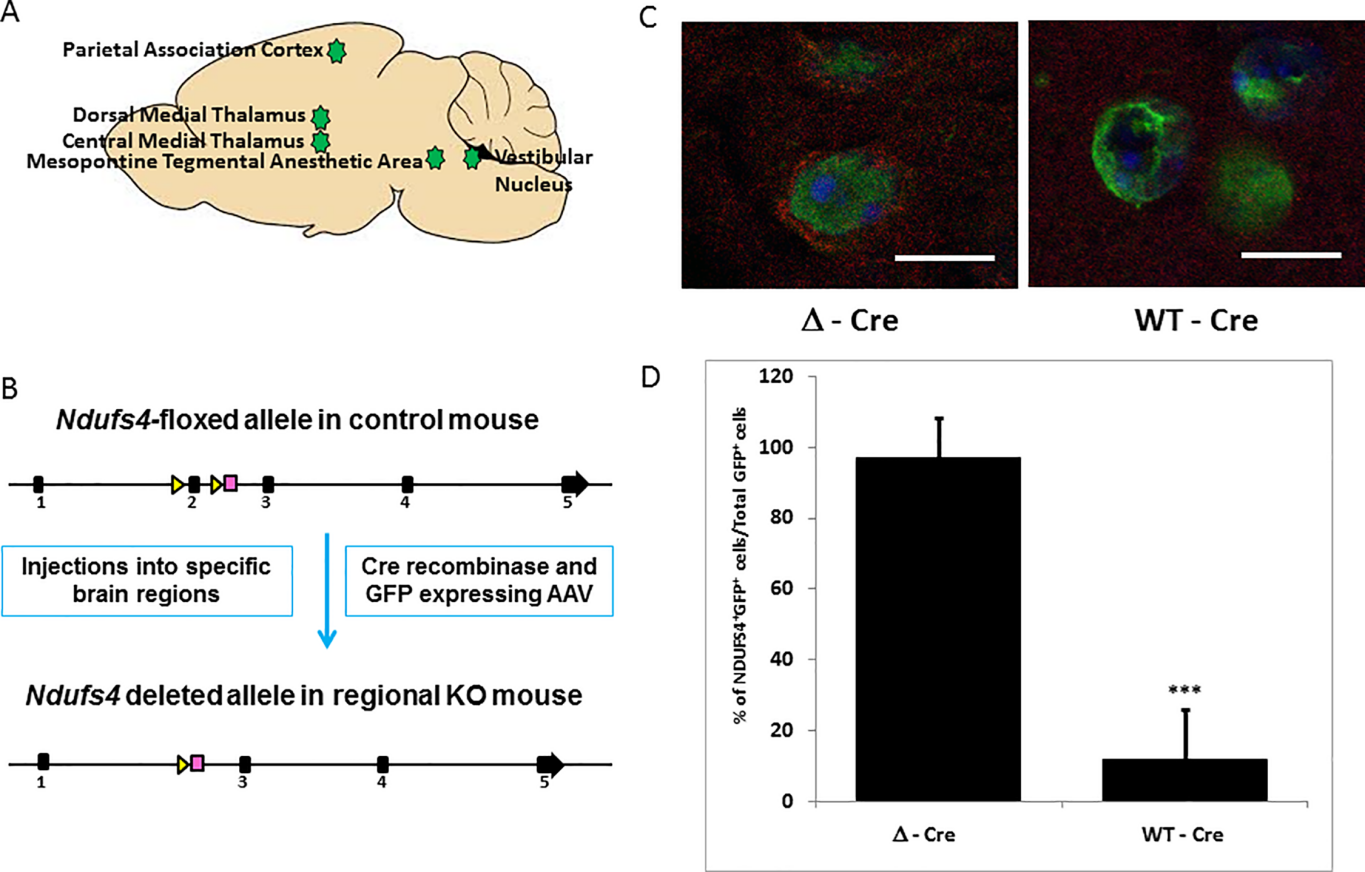
Viral injections and characterization. **A**. Schematic diagram depicting the five regions which were injected with either rAAV/WT-Cre GFP (knockout virus expressing functional Cre recombinase) or rAAV/Δ-Cre GFP (sham virus expressing nonfunctional Cre recombinase). **B**. Schematic showing the mechanism of gene deletion. Exon 2 of genomic *Ndufs4* is excised by the virus expressing active Cre recombinase, resulting in the loss of NDUFS4 protein as described by Kruse *et al*. [6]. **C**. Loss of *Ndufs4* expression in active Cre virus infected cells in the PAC. Infected cells appear green under the confocal microscope due to the viral GFP co-expression (Magnification X1000). In the Δ-Cre sham virus infected cells of the PAC, mitochondrial NDUFS4 fluorescence (red) is seen, which is absent in the WT-Cre infected cells. **D**. Representative quantitation of NDUFS4 expression in the virus infected cells, shown as a percentage of total infected cells. 20 image fields were quantified per injected locus. Scale bar: 10μm. *** indicates p-values <0.001.

We previously reported the striking hypersensitivity of *Ndufs4* global KO mice to ISO and HAL for the TC assay (S1 Table) [3]. Here, we measured the change in sensitivity of the global KO for loss of righting reflex (LORR). *Ndufs4(KO)* mice were hypersensitive to ISO (EC_50_(control) 0.96±0.08%, EC_50_(KO) 0.42±0.08%; p = 3.1X10^-7^) and HAL (EC_50_(control) 0.95±0.03%, EC_50_(KO) 0.41±0.05%; p = 1.4X10^-10^) (S1 Table). Active (WT-Cre) and inactive (Δ-Cre) viruses, each linked to a GFP construct to establish localization, were then injected into five regions of the CNS of *Ndufs4*^lox/lox^ mice (Fig 1A and 1B). Since the half-life of NDUFS4 is around 17 days [21], we performed behavioral testing and histological analyses three weeks after viral injections. Robust knockdown of *Ndufs4* in the regions injected with WT-Cre virus was confirmed by immunohistochemistry (Fig 1C, S1 Fig) at 4 weeks post virus injections. The loss of NDUFS4 immunostaining in the GFP-expressing virus infected cells in the injected regions was quantified to confirm virus mediated knockdown of NDUFS4 (Fig 1D). We found that cells near the periphery of the WT-Cre injected foci, which did not take up the virus, retained the expression of NDUFS4 (S1B Fig). By immunostaining, we found that cytochrome C expression, indicating the presence of mitochondria, remained unaltered in the WT-Cre and Δ-Cre injected regions (S2 Fig).

### Vestibular nucleus (VN)

**Fig 2:**
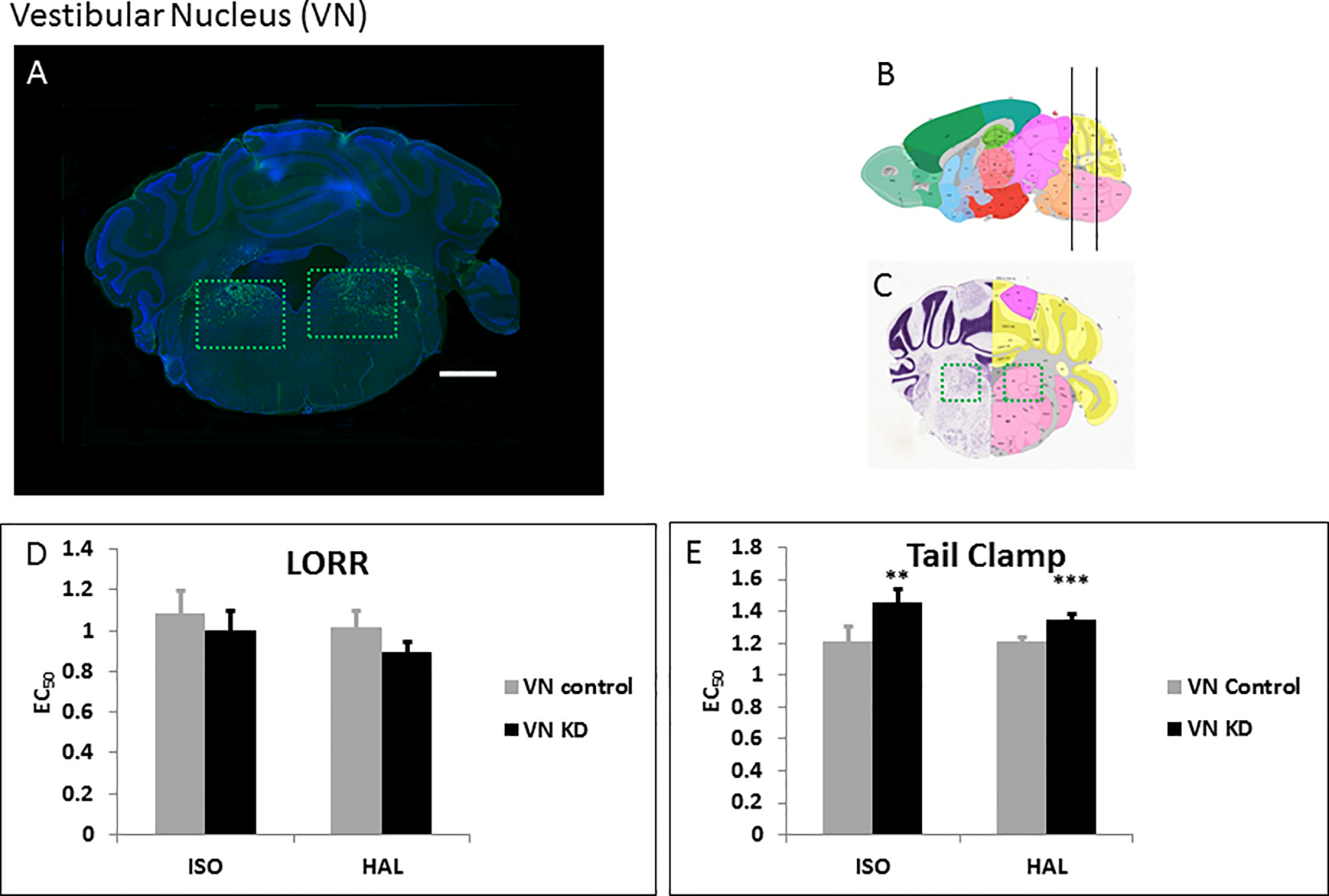
Knockdown of *Ndufs4* in the VN. **A.** Fluorescent images of brain slices from mice injected with active WT-Cre virus into the VN (Coordinates: ML = ± 1.25; AP = -5.8; DV = 4.3, Magnification X40). Virus-infected cells appear green due to the viral GFP co-expression B, **C.** Schematic figures from the Allen mouse brain atlas [24] (Image credit: Allen Institute) depicting the antero-posterior (**B**), mediolateral (**C**) and dorso-ventral (**C**) viral spread (green boxed regions in **C**). Reprinted from the Allen mouse brain atlas under a CC BY license, with permission from the Allen Institute, original copyright 2008. **D.** EC_50_s for ISO and HAL for the active (n = 7, black bars) and sham (n = 6, grey bars) virus injected mice in the LORR assay. **E.** EC_50_s for ISO and HAL for the active (n = 7, black bars) and sham (n = 6, grey bars) virus injected mice in the TC assay. Scale bar: 1mm. **indicates p-values <0.005, *** indicates p-values <0.001. The error bars in all bar graphs indicate standard deviation.

The VN is one of the regions showing maximal pathology in *Ndufs4(KO)* animals and is implicated in locomotor ability and nociceptive signaling [22, 23]. We injected WT-Cre (Fig 2A, 2B and 2C) and Δ-Cre (S3A Fig) bilaterally into the VN. Loss of *Ndufs4* in the VN decreased sensitivity of the mice and caused resistance to both ISO and HAL for TC (Fig 2E, S4B Fig; Table 1). There was no statistically significant difference in the LORR between VN specific KD and control animals for either anesthetic (Fig 2D, S4A Fig; Table 1).

**Table 1:** EC_50_s for ISO and HAL for the WT-Cre and Δ-Cre virus-injected mice in the LORR and TC assays.

Type of injection	Region injected	Behavioral test	EC_50_ ISO (SD)	EC_50_ HAL (SD)	N (Gender)
AAV-WT-Cre-GFP	VN	LORR	1.00 (0.09)	0.90 (0.05)	7 (4F/3M)
AAV-Δ-Cre-GFP	VN	LORR	1.1 (0.1)	1.02 (0.08)	6 (4F/2M)
AAV-WT-Cre-GFP	VN	TC	1.45 (0.09)* p = 0.002	1.35 (0.04)* p = 3.6X10^-5^	7 (4F/3M)
AAV-Δ-Cre-GFP	VN	TC	1.2 (0.1)	1.21 (0.02)	6 (4F/2M)
AAV-WT-Cre-GFP	MPTA	LORR	0.79 (0.06)* p = 0.01	0.82 (0.06)	6 (3F/3M)
AAV-Δ-Cre-GFP	MPTA	LORR	0.90 (0.07)	0.87 (0.04)	7 (3F/4M)
AAV-WT-Cre-GFP	MPTA	TC	1.1(0.2)	1.1 (0.1)	6 (3F/3M)
AAV-Δ-Cre-GFP	MPTA	TC	1.25 (0.05)	1.23 (0.09)	7 (3F/4M)
AAV-WT-Cre-GFP	CMT	LORR	0.80 (0.07)* p = 0.003	0.77 (0.07)* p = 0.004	6 (4F/2M)
AAV-Δ-Cre-GFP	CMT	LORR	1.0 (0.1)	1.0 (0.1)	6 (2F/4M)
AAV-WT-Cre-GFP	CMT	TC	1.0 (0.1)* p = 0.008	1.02 (0.08)* p = 3.4X10^-5^	6 (4F/2M)
AAV-Δ-Cre-GFP	CMT	TC	1.2 (0.1)	1.31 (0.08)	6 (2F/4M)
AAV-WT-Cre-GFP	DMT	LORR	0.80 (0.03)* p = 0.001	0.74 (0.02)* p = 3.8X10^-7^	6 (4F/2M)
AAV-Δ-Cre-GFP	DMT	LORR	0.93 (0.04)	1.02 (0.05)	6 (2F/4M)
AAV-WT-Cre-GFP	DMT	TC	0.92 (0.09)* p = 0.0004	0.87 (0.04)* p = 1.2X10^-7^	6 (4F/2M)
AAV-Δ-Cre-GFP	DMT	TC	1.2 (0.1)	1.33 (0.07)	6 (2F/4M)
AAV-WT-Cre-GFP	PAC	LORR	0.71 (0.05)* p = 3.5X10^-7^	0.68 (0.06)* p = 0.0001	6 (1F/5M)
AAV-Δ-Cre-GFP	PAC	LORR	0.95 (0.02)	1.0 (0.1)	6 (3F/3M)
AAV-WT-Cre-GFP	PAC	TC	0.85 (0.05)* p = 9.4X10^-8^	0.77 (0.09)* p = 5.4X10^-7^	6 (1F/5M)
AAV-Δ-Cre-GFP	PAC	TC	1.21 (0.06)	1.23 (0.07)	6 (3F/3M)

* indicates p-values < 0.01. p values listed compare region specific KO EC_50_s to control animals injected with sham Cre.

### Mesopontine tegmental area (MPTA)

**Fig 3:**
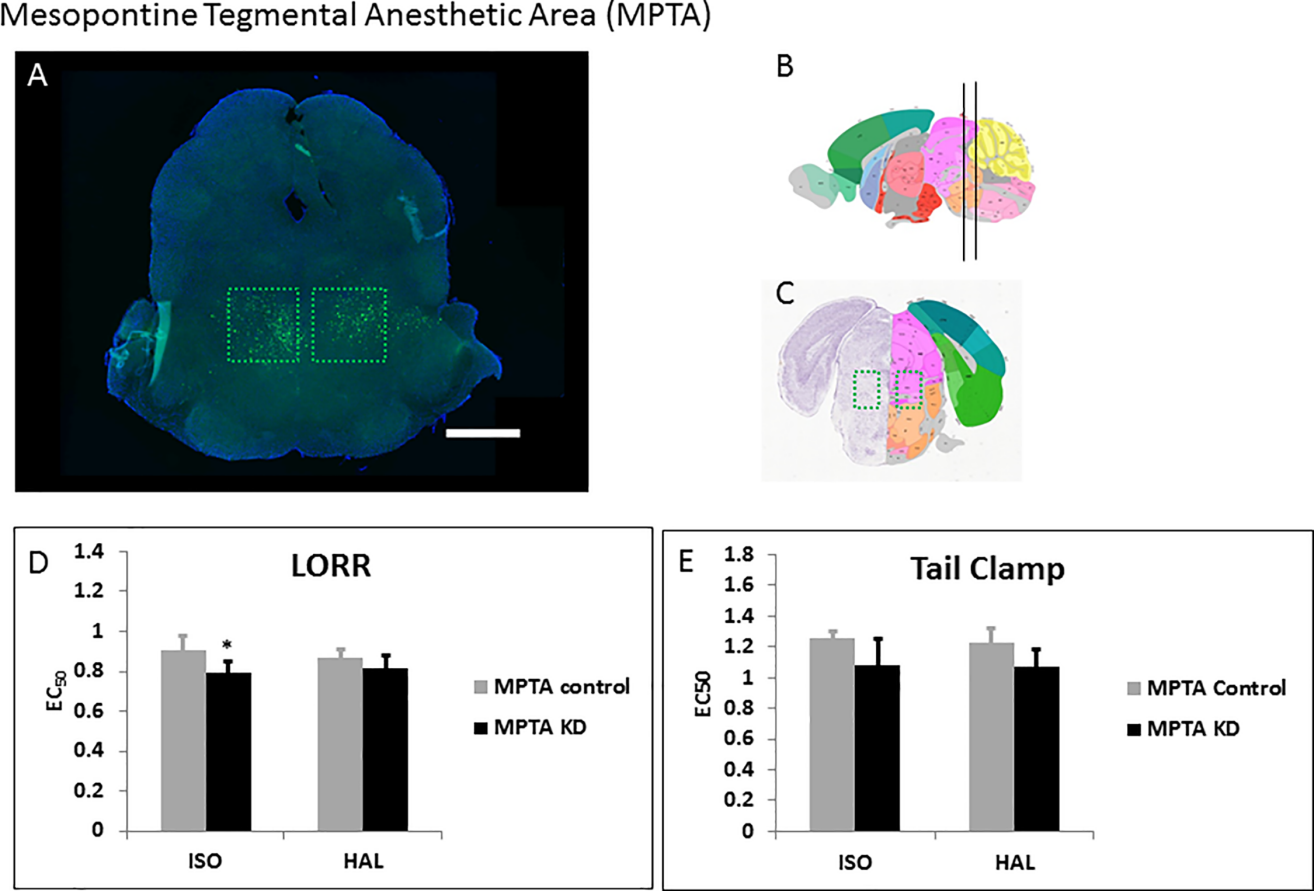
Knockdown of *Ndufs4* in the MPTA. **A.** Fluorescent images of brain slices from mice injected with active WT-Cre virus (**A**) into the MPTA (Coordinates: ML = ± 0.70; AP = -4.6; DV = 4.1, Magnification X40). **B**, **C.** Schematic figures from the Allen mouse brain atlas [24] depicting the viral spread (Image credit: Allen Institute). Reprinted from the Allen mouse brain atlas under a CC BY license, with permission from the Allen Institute, original copyright 2008. **D.** EC_50_s for ISO and HAL for the active (n = 6, black bars) and sham (n = 7, grey bars) virus injected mice in the LORR assay. **E.** EC_50_s for ISO and HAL for the active (n = 6, black bars) and sham (n = 7, grey bars) virus injected mice in the TC assay. Scale bar: 1mm. **indicates p-value <0.01.

Based on the data of Devor and colleagues using rats [17], we followed anatomical co-ordinates for mice and injected the WT-Cre (Fig 3A, 3B and 3C) and Δ-Cre viruses (S3B Fig) bilaterally into the calculated mouse brain MPTA. There was no statistically significant change in the TC response of MPTA specific KD to either anesthetic when compared to the sham virus injected group (Fig 3E, S4D Fig; Table 1). While the KDs did not show any change in sensitivity to HAL for LORR, they showed a statistically significant increase in sensitivity to ISO for LORR (Fig 3D, S4C Fig; Table 1).

### Central medial thalamus (CMT)

**Fig 4:**
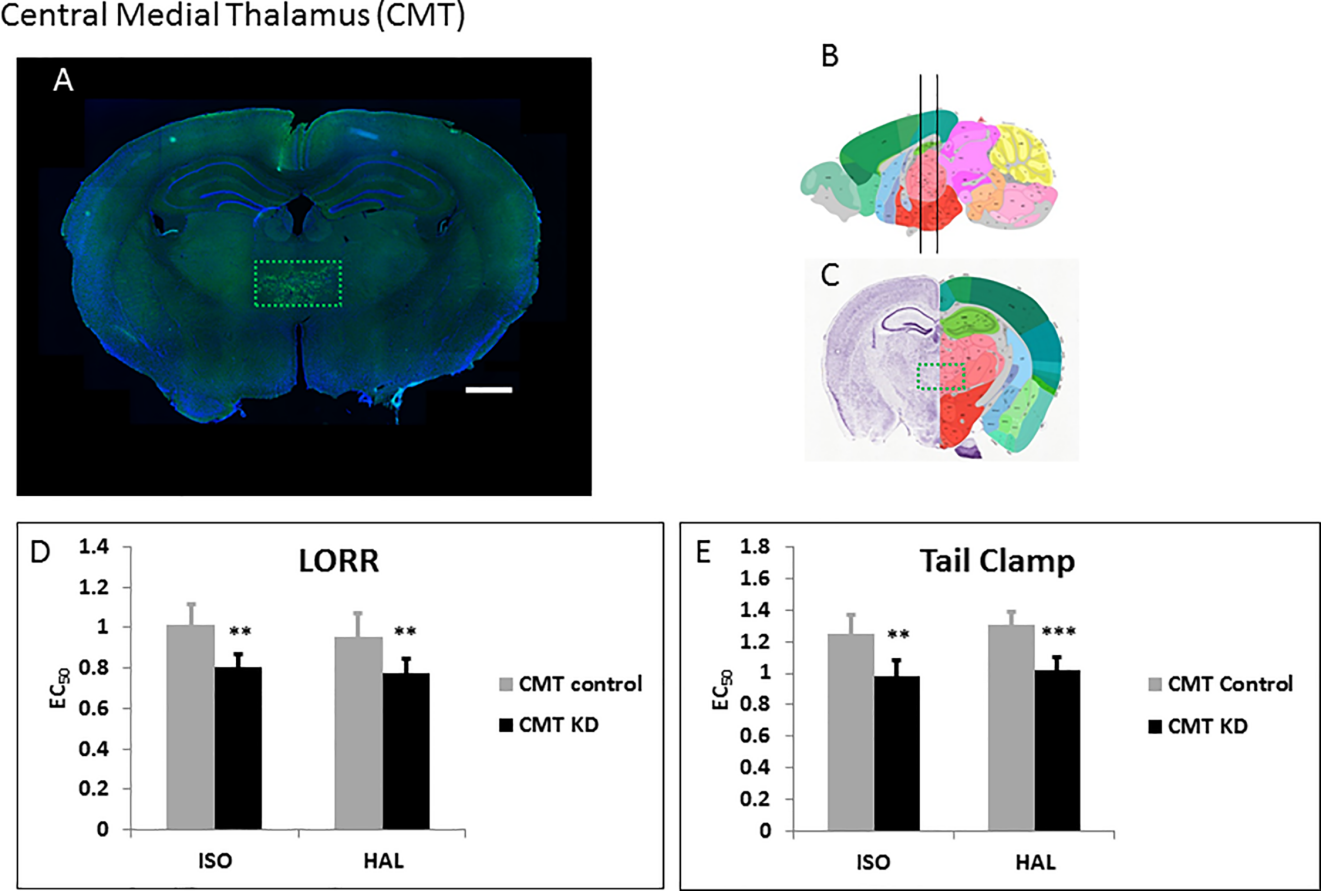
Knockdown of *Ndufs4* in the CMT. **A.** Fluorescent images of brain slices from mice injected with active WT-Cre virus (**A**) into the CMT (Co-ordinates: ML = ± 0.32; AP = -1.2; DV = 3.85, Magnification X40). **B**, **C.** Schematic figures from the Allen mouse brain atlas [24] depicting the viral spread (Image credit: Allen Institute). Reprinted from the Allen mouse brain atlas under a CC BY license, with permission from the Allen Institute, original copyright 2008. **D.** EC_50_s for ISO and HAL for the active (n = 6, black bars) and sham (n = 6, grey bars) virus injected mice in the LORR assay. **E.** EC_50_s for ISO and HAL for the active (n = 6, black bars) and sham (n = 6, grey bars) virus injected mice in the TC assay. Scale bar: 1mm. **indicates p-values <0.005, *** indicates p-values <0.001.

The CMT is critically involved in sensory relaying to the cortex; this region has been shown to play roles in anesthesia [10, 25] and sleep mediation [26]. We injected the WT-Cre (Fig 4A, 4B and 4C) and Δ-Cre (S3C Fig) viruses bilaterally into the CMT. Knocking down *Ndufs4* in the CMT caused hypersensitivity to both ISO and HAL for TC (Fig 4E, S4F Fig; Table 1). Similarly for LORR, loss of *Ndufs4* in the CMT made mice hypersensitive to ISO and HAL (Fig 4D, S4E Fig; Table 1).

### Dorsal medial thalamus (DMT)

**Fig 5:**
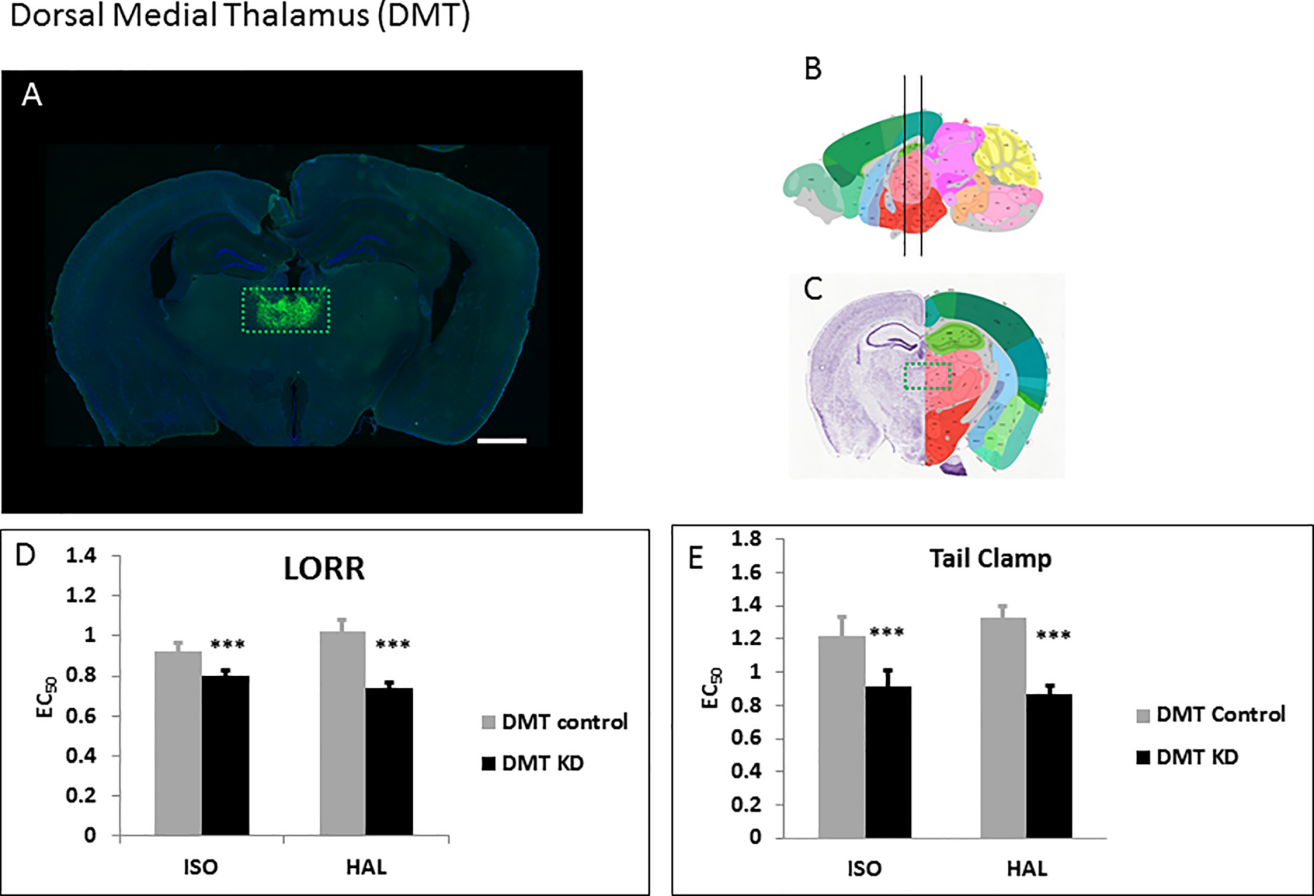
Knockdown of *Ndufs4* in the DMT. **A.** Fluorescent images of brain slices from mice injected with active WT-Cre virus (**A**) into the DMT (Co-ordinates: ML = ± 0.32; AP = -1.2; DV = 3.7, Magnification X40). **B**, **C.** Schematic figures from the Allen mouse brain atlas [24] depicting the viral spread (Image credit: Allen Institute). Reprinted from the Allen mouse brain atlas under a CC BY license, with permission from the Allen Institute, original copyright 2008. **D.** EC_50_s for ISO and HAL for the active (n = 6, black bars) and sham (n = 6, grey bars) virus injected mice in the LORR assay. **E.** EC_50_s for ISO and HAL for the active (n = 6, black bars) and sham (n = 6, grey bars) virus injected mice in the TC assay. Scale bar: 1mm. *** indicates p-values <0.001.

The DMT has been implicated in mediolateral pain modulation and anesthesia mediation by pentobarbital [27]. We injected the WT-Cre (Fig 5A, 5B and 5C) and Δ-Cre (S3D Fig) viruses bilaterally into the DMT. Removal of *Ndufs4* caused significant hypersensitivity to both ISO and HALfor TC (Fig 5E, S4H Fig; Table 1). Similarly for LORR, loss of *Ndufs4* in the DMT made mice hypersensitive to ISO and HAL (Fig 5D, S4G Fig; Table 1).

### Parietal association cortex (PAC)

**Fig 6:**
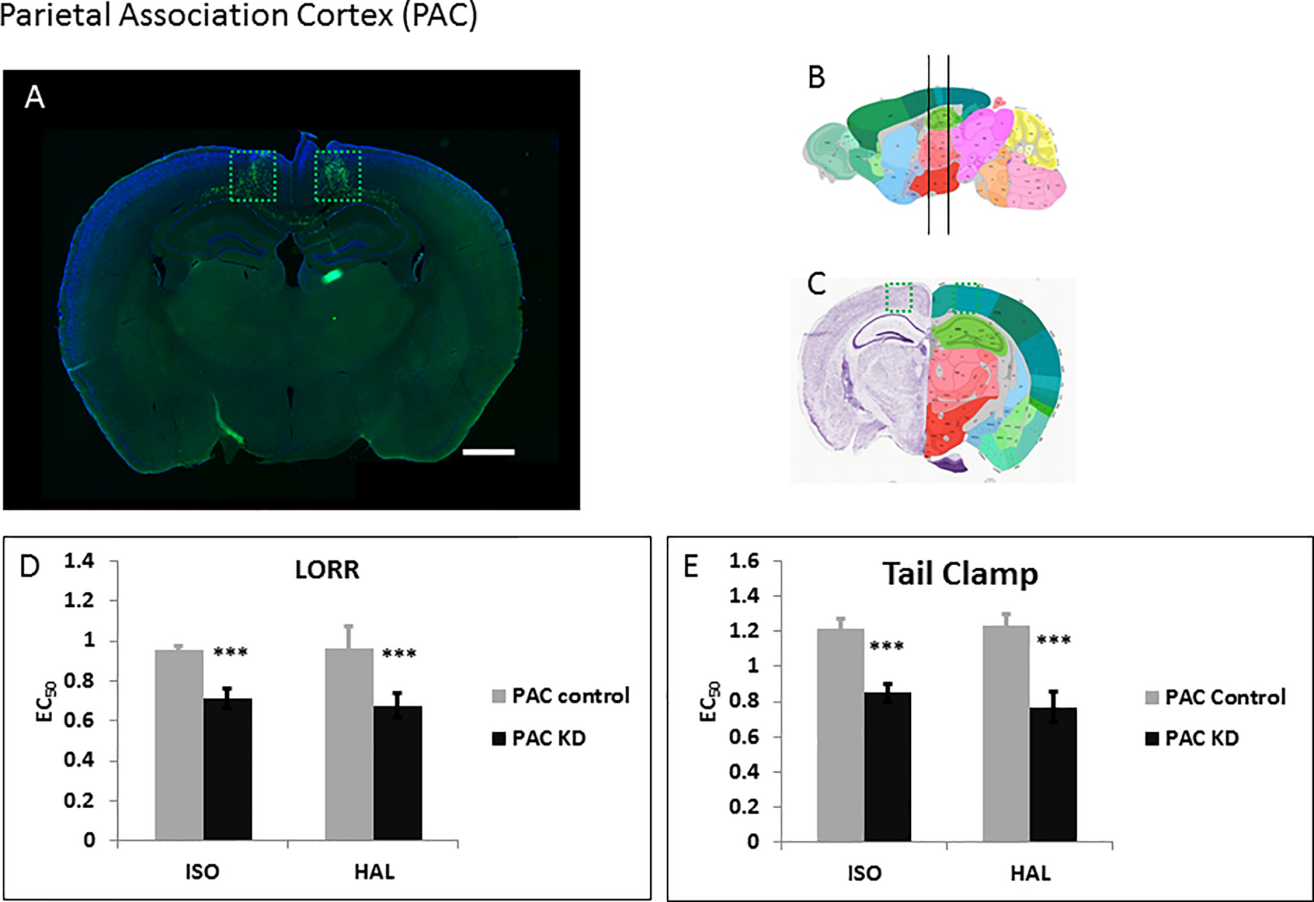
Knockdown of *Ndufs4* in the PAC. **A.** Fluorescent images of brain slices from mice injected with active WT-Cre virus (**A**) into the PAC (Co-ordinates: ML = ± 0.80; AP = -1.75; DV = 0.9, Magnification X40). **B**, **C.** Schematic figures from the Allen mouse brain atlas [24] depicting the viral spread (Image credit: Allen Institute). Reprinted from the Allen mouse brain atlas under a CC BY license, with permission from the Allen Institute, original copyright 2008. **D.** EC_50_s for ISO and HAL for the active (n = 5, black bars) and sham (n = 6, grey bars) virus injected mice in the LORR assay. **E.** EC_50_s for ISO and HAL for the active (n = 5, black bars) and sham (n = 6, grey bars) virus injected mice in the TC assay. Scale bar: 1mm. *** indicates p-values <0.001.

The CMT and DMT neurons send major projections into the posterior PAC (Allen Mouse Brain Connectivity Database) [28]. The PAC is involved in episodic memory retrieval, spatial reasoning, perception and attention. We surmised that glutamatergic relaying to the cortex might be compromised in the regional knockdown, leading to hypersensitivity to anesthetics. We therefore injected the WT-Cre (Fig 6A, 6B and 6C) and Δ-Cre (S3E Fig) viruses bilaterally into the PAC. Knock down of *Ndufs4* in the PAC caused the highest level of hypersensitivity recorded in our study for both ISO and HAL for TC (Fig 6E, S4J Fig; Table 1). For LORR, knockdown of *Ndufs4* in the PAC caused hypersensitivity to ISO and HAL (EC_50_(control) 1.0±0.1%, EC_50_(KD) 0.68±0.06%; p = 0.0001) (Fig 6D, S4I Fig; Table 1).

**Fig 7:**
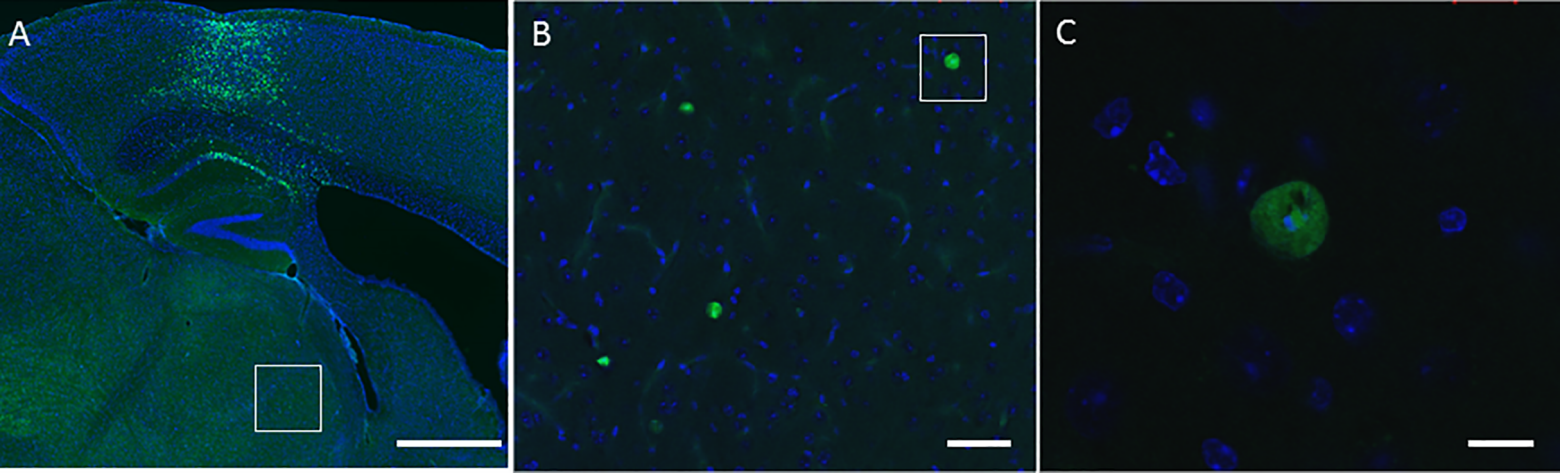
The thalamocortical circuit. **A.** Fluorescent image of a brain slice from a mouse injected with Δ-Cre virus into the PAC (Magnification X40). **B.** Magnified confocal image of the white-boxed region within the thalamus in **A** (Magnification X200)**. C.** Magnified confocal image of white boxed region in **B** showing viral-GFP expressing cells in the thalamus following injection into the PAC (Magnification X1000). Scale bars: A-1mm, B-50μm, C-10μm.

**Fig 8:**
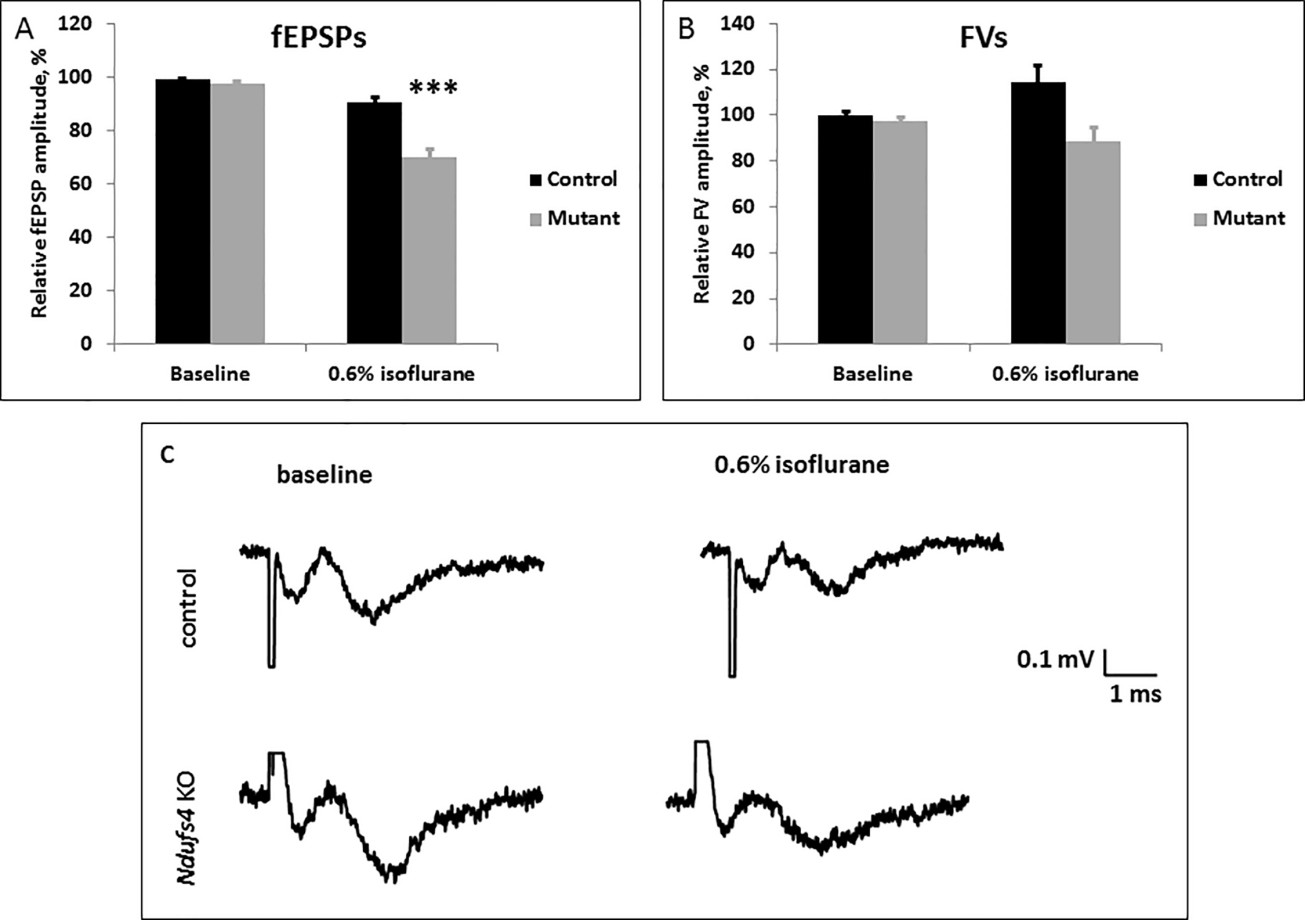
Electrophysiological field recordings of the excitatory synaptic transmission. **A.** Amplitudes of fEPSPs decreased with 0.6% (248 μM) ISO exposure in control and *Ndufs4(KO)* brain slices. The decrease of fEPSPs was significantly greater for the *Ndufs4(KO)* than the control (*** indicates p-value <0.001). **B.** Fiber volleys of both genotypes were not significantly affected by the isoflurane exposure. **C.** Representative traces of field recordings from control and KO slices before and during exposure to 0.6% ISO (equivalent to 248 μM). 0.6% ISO exposure led to a significantly larger decrease in the amplitudes of fEPSP in the KO when compared to the respective decrease in controls. The amplitudes of the fiber volleys were decreased (statistically tending to significance, p = 0.015) by 0.6% ISO exposure in both genotypes.

Brightly fluorescing cells also appeared in the central thalamus of the brains of mice injected with the Δ–Cre and WT-Cre viruses (Fig 7) into the PAC. This provides evidence of anterograde transmission of the GFP signal from the PAC to the thalamus and for the existence of a thalamocortical circuit possibly involved in mediating the anesthetic sensitivity of the *Ndufs4(KO)* mice [3]. To determine whether the excitatory neurotransmission in the PAC circuit is compromised by *Ndufs4* mutation, we recorded the field excitatory postsynaptic potentials (fEPSPs) in the PAC of sagittal brain slices of *Ndufs4(KO)* and control sibling mice (Fig 8). The amplitudes of fEPSPs in the PAC were significantly decreased during 0.6% ISO exposure (corresponds to 248 μM at room temperature) in the *Ndufs4(KO)* when compared to the control slices (p = 0.00008). There was a trend for decrease in the fiber volley amplitude of the global KO brain slices during 0.6% ISO exposure when compared to the control brain slices, however the change did not reach statistical significance (p = 0.015).

## Discussion

Inactivation of a complex I subunit in the thalamocortical circuit caused striking VA hypersensitivity to both the LORR and TC responses. The degrees of change are especially noteworthy given that inactivation specifically knocked down levels of the protein only in very confined regions of the CNS, after development was largely completed. Acute local inactivation of a mitochondrial gene in an otherwise wild type mouse minimizes compensatory developmental changes or latent nonspecific CNS degeneration seen in the global KO, as potential confounders of data interpretation. Although we did not measure mitochondrial function in these microscopic regions of the brain, we infer that complex I function is defective in the regions where *Ndufs4* is knocked down. In turn, we interpret these data to indicate that anesthetic hypersensitivity here results specifically from acute mitochondrial dysfunction. The simplest explanation of our data is that VAs directly inhibit complex I function [29]; the resulting region-specific decrease in metabolism leads to the anesthetized state.

Regional metabolism is certainly altered in the CNS while anesthetized, but this may result from or cause the anesthetized state [19, 25, 30]. However, we know that defects in mitochondrial complex I increase anesthetic sensitivity without known changes in ion channel characteristics [9]. In addition, the EC_50_ of the *Ndufs4(KO)* mutant, 0.4% isoflurane, is well below the concentration that produces significant effects in putative VA targets that may reduce regional metabolism as a secondary effect. Given that complex I dysfunction profoundly sensitizes worms, mice and children to VAs [1–3, 31], this effect appears highly conserved across the animal kingdom.

Mitochondrial function represents a key component of normal CNS metabolism, since oxidative phosphorylation is necessary for synaptic function [32, 33]. Complex I is the rate limiting step of electron transport [34, 35]; isolated mitochondria from the *Ndufs4(KO)* brain display significantly reduced Complex I activity [7]. Prior hypotheses suggested a generalized decrease in neuronal activity as a mechanism of anesthesia [10, 12]. However, recent work indicates that localized CNS effects may underlie the anesthetic state and multiple regions have been suggested as mediating these effects [14, 19, 30, 36, 37]. The data presented here highlight the probable importance of normal mitochondrial function within very circumscribed key regions of the brain that are necessary to support normal response to anesthetics. The anesthetic inhibition of response to TC is thought to result from effects on the spinal cord with some supraspinal modification [18, 38]. The LORR is used as a surrogate for unconsciousness [39, 40]. Our results demonstrate different phenotypes for these endpoints mediated by mitochondrial dysfunction in various regions of the brain. We discuss LORR and TC separately below.

## Loss of righting reflex

It is known that the thalamus displays suppressed metabolism [19] and decreased synaptic activity during anesthesia [37, 41]. The hypersensitivity seen in our animals after viral injection into the medial thalamus corroborates a role for the medial thalamic nuclei in determining anesthetic sensitivity for LORR. Our results support electrophysiologic work showing thalamocortical activity correlated with anesthetic depth [42]. Since the medial thalamus relays glutamatergic signals to the cortex [43, 44], VAs likely exert their effects through suppression of these pathways. The mouse connectivity database of the Allen Institute also shows projections from the central medial thalamus populating the parietal association cortex [28, 45]. We have confirmed a circuit between the PAC and the thalamus, with reciprocal projections from the PAC to the thalamus. Viral inactivation of *Ndufs4* in the PAC reveals that this region contributes about 1/2 of the hypersensitivity displayed by the whole body *Ndufs4(KO)* for ISO and HAL in the LORR assay (Table 1).

The reduced fEPSP amplitudes in the PAC of *Ndufs4* global KO during 0.6% ISO exposure likely contributes to the increased VA sensitivity seen in the global KO. This is consistent with studies demonstrating that dexmedetomidine induced loss of consciousness in healthy volunteers preferentially decreased cerebral glucose metabolic rate and blood flow in thalamus and in an area corresponding to the PAC of mice [19]. While the functional connectivity within cortical regions was preserved, connectivity between medial thalamus and the cortical regions was disrupted during the unconscious state and re-established during regain of consciousness. This same area, part of a larger region called the DMN (default mode network) in humans [46], shows a direct correlation between connectivity within the DMN and levels of consciousness in patients with disorders of consciousness [47, 48]. We conclude that the thalamocortical sensory relaying is bioenergetically demanding and sensitive to mitochondrial inhibition by VAs.

Studies by Devor and colleagues in rats have shown that the mesopontine tegmental anesthetic area (MPTA) is important for mediating anesthetic LORR sensitivity to pentobarbital [17]. However, we found MPTA KD mice were mildly hypersensitive only to ISO for LORR. It is possible that our injections did not localize to the MPTA, or the precise location of the MPTA may differ between the rat and mouse brain. It is also possible that the MPTA is not an important region for mediating the anesthetic sensitivity of HAL, or that the mitochondrial mutation mediates anesthetic hypersensitivity by exerting regional effects elsewhere.

## Tail clamp

Similar to the effects on LORR, *Ndufs4* loss in the CMT/DMT and PAC accounts for approximately half of the hypersensitivity displayed by the whole body KO for ISO and HAL in the TC assay [3]. The ascending spinal cord and the ventro-posterior brain stem nuclei of the primary somatic pathways transmit nociceptive signals from the peripheral nervous system to the somatosensory, the parieto-insular and the anterior cingulate cortices resulting in pain perception [49, 50]. The medial thalamus is involved in the medial pain perception pathway and is suppressed by pentobarbital during noxious stimulation [27]. The DMT also drives feed forward inhibition of the anterior cingulate cortex to limit excitatory input processing and firing of cortical neurons, resulting in better temporal precision of the circuit [51]. Similar to the effects on LORR, we propose that mitochondrial dysfunction disrupts the temporal fidelity within the thalamocortical circuits such that the pain perception pathway is inhibited more completely by ISO and HAL when mitochondrial dysfunction is present prior to anesthetic exposure.

It is interesting that VN-specific *Ndufs4* KD caused resistance to VAs in the tail clamp assay. In contrast to the role played by the cortex in central pain perception, the higher brain centers have also been shown to modulate nociceptive transmission so that spinal transection increases nociceptive reflexes [52]. The descending spinal cord modulatory effects by the higher brain centers could be facilitating or inhibitory [53, 54]. Antognini *et al*. showed that preferential anesthetization of the goat brain bypassing the spinal cord increased the isoflurane requirement to inhibit the sensitivity to dew-claw stimulation [18]. Rampil *et al*. showed that depression of the spinal motor neurons may contribute to the lack of response to painful stimuli mediated by various inhalational anesthetics in rats [55, 56]. Our results are most consistent with descending inhibitory signaling through the VN to the spinal cord being decreased by the *Ndufs4(KO)*.

We propose that the excitatory thalamocortical firing to the PAC mediates both consciousness and nociception. The posterior brain stem acts as a switch between nociception, during which excitatory cortical firing to the brain stem dominates, and antinociception, during which inhibitory cortical firing to the VN and the downstream spinal neurons dominates. Supporting the model, glucose uptake [57] and c-FOS staining [58] in the VN increased during isoflurane anesthesia, indicating that the anesthetized state may require increased energy production in the VN which is rendered inadequate by the mitochondrial mutation.

## Conclusion

We report the first demonstration linking regional CNS inactivation of a mitochondrial protein to sensitivity to volatile anesthetics. We infer that localized knockdown of NDUFS4 results in a regional defect in mitochondrial respiration as is seen in the global KO. Mitochondria therefore represent a putative novel target for volatile anesthetics. Restriction of mitochondrial defects to the thalamocortical circuit causes greater than half of the change in anesthetic sensitivities seen in the total loss of the *Ndufs4* gene. Regional bioenergetic capacities and mitochondrial function are likely an important determinant of response to volatile anesthetics, and may underlie the mechanism of action of volatile anesthetics.

## Materials and methods

### Animal maintenance

All studies were approved by the Animal Care and Use Committee of the Seattle Children’s Research Institute. Mice were maintained on standard rodent diet at 22°C with 12-hour dark/light cycles. *Ndufs4*^lox/lox^ mice with a mixed 129/Sv:C57Bl/6 genetic background [6] were a kind gift from Richard Palmiter (Howard Hughes Medical Institute and the University of Washington, Seattle, WA).

### Intracranial virus delivery

The adeno-associated viruses rAAV/WT-Cre GFP and the rAAV/Delta(Δ)-Cre GFP viruses [59, 60] were purchased from UNC Vector Core (Chapel Hill, NC) and diluted in PBS at a titer of 2 × 10^9^ viral genomes/μl. These viruses encode wild-type active Cre-recombinase (WT-Cre, active) or mutated defective Cre-recombinase (Δ-Cre, control) respectively and green fluorescent protein (GFP) and are known to be taken up by all types of cells in the injected region [61]. Delivery of active Cre expressing AAV to the various regions of *Ndufs4*^lox/lox^ mice brains created region-specific loss of the protein NDUFS4. Male and female animals aged between 35 to 40 days of age were anesthetized with isoflurane and either virus was injected into various brain regions using the motorized Robot stereotax (Neurostar GmbH, Tubingen, Germany) as per manufacturer instructions. Briefly, the anesthetized mouse was attached to the stereotax with inline isoflurane (induction with 5% ISO and maintenance with 1.5% ISO) delivered through a face mask. An incision was made on the shaved and sterilized supracranial skin exposing the skull. Either virus was injected using a motorized injector after drilling holes in the cranium at stereotaxically defined bilateral locations. The quantities and co-ordinates of the viral injections are listed in S2 Table. For VN, CMT, DMT and PAC, coordinates were used from the Allen Brain Atlas. The location of the MPTA was approximated by using rat coordinates. The mice were allowed to recover for 3 weeks before behavioral testing using halothane (HAL) or isoflurane (ISO).

### Behavioral testing

Mice were anesthetized with ISO or HAL and assayed for LORR or response to TC as described by Sonner *et al*. [62] and the anesthetic concentrations analyzed by gas chromatography [63]. The EC_50_s for loss of response to TC were used as the equivalent of minimum anesthetic concentrations (MAC) for loss of nociceptive motor response. The EC_50_s for the loss of righting reflex were used as a measure of the consciousness status of the animal. Animals were kept warm on a heating pad throughout and allowed to recover for at least 24 h between behavioral assays. Each animal underwent TC and LORR testing in a randomized order. No significant differences were noted between the anesthetic sensitivity of the subjected mice due to prior anesthetic exposure during the randomized tests. The EC_50_ for an anesthetic was the average of the concentrations for induction (defined as the average concentrations of gas in the last sample before loss of response and in the first sample at which the response was lost) and emergence (defined as the average concentration of gas in the first sample at which the animal once again responded, and the sample immediately prior). Mice were tested at 3 weeks after virus injection for both behavioral endpoints in both anesthetics. All animals survived the anesthetic exposures. They were then sacrificed for immunohistochemical analyses at 28 days after injection.

### Histopathological analyses

Mice were euthanized using CO_2_, and brains were fixed in cold 4% paraformaldehyde, postfixed overnight at 4°C, cryoprotected in 30% sucrose-PBS for three days and embedded in optimal cutting temperature compound (OCT). Brains were then sliced at 30 μm thickness and serial slices were collected in PBS in 24-well dishes. Slices at 180μm intervals were selected and mounted on glass slides, analyzed and imaged under a fluorescence microscope (upright Zeiss Axioskop equipped with an Attoarc 100W HBO lamp, an Axiocam color charge-coupled device camera (type 412–312) and Axiovision v3.1 software) to assess the viral spread of infection. For heat-induced epitope retrieval, captured slices on glass slides were boiled in 10mM sodium citrate buffer (pH 6.0) at 100°C for 20 minutes using a water bath [64]. Slides were then blocked in PBS with 10% normal donkey serum overnight at 4°C. Primary antibodies, mouse anti-NDUFS4 (Santa Cruz; dilution 1:50), mouse anti-cytochrome C (MitoSciences; dilution 1:300) and goat anti-*GFP* (Abcam; dilution 1:200) were incubated overnight at 4°C. Secondary antibodies, rabbit anti-mouse Alexa Fluor 568 (Life Technologies; dilution 1:2000) and donkey anti-goat AlexaFluor 488 (Life Technologies; dilution 1:2000) were incubated for one hour at RT. After mounting the slices in DAPI Fluoromount-G solution (Southern Biotech), they were imaged by confocal microscopy (Zeiss LSM 710 Imager Z2 laser scanning confocal microscope, Zen 2009 software). To assess the efficiency of the AAV-mediated knockdown, Alexa Fluor 568 tagged NDUFS4 expression (red immunofluorescence) was quantified in the virus infected cells which co-expressed the Green Fluorescent Protein (GFP, Alexa Fluor 488 tagged, green immunofluorescence), and expressed as a percentage of total infected cells. 20 image fields were quantified per injected locus. These were from 5 bilateral alternating slices, 4 image fields per slice, of 85μmX85μm size each, quantified by a blinded scorer.

### Field potential recordings

Field potential experiments were performed as described previously with modifications [9]. Briefly, 400 μm thick sagittal slices of the mouse brain were collected. The recording electrode was placed in parietal association cortex and the stimulation electrode was positioned ~1mm rostrally in the cortex. The amplitude was selected to produce half-maximal fEPSP amplitude. The stimulus duration was 100 μs. Field potential signals were amplified using EPC10 amplifier (HEKA) and digitized with Digidata 1322A (Axon Instruments) at 250 kHz sampling rate. Stimulations were applied at 0.033 Hz. Isoflurane was applied in the superfusate at equilibrated concentrations delivered by passing carbogen through a calibrated isoflurane vaporizer (Scivena Scientific TEC-3 and Patterson Veterinary TEC-3). Amplitudes of fEPSPs and FVs were normalized to their respective averages during the last 5 min prior to isoflurane exposure for each individual recording.

### Statistical analyses

A statistical power analysis was performed using the control values and standard deviation for the uninjected control mice as reference. Specifying an effect size of 0.25, α of 0.05 and power of 0.8 for each assay (LORR-ISO, LORR-HAL, TC-ISO and TC-HAL), we found the minimal sample size to be 2. For all region-specific injections, we used a minimal cohort size of 6 per category. The effective concentration for 50% of the animals tested (EC_50_) for each volatile anesthetic was determined as described by Sonner *et al*. [62], using an up and down method. Values for EC_50_s were compared between the WT and regional KD strains by Student’s t-test using GraphPad Prism®. For the electrophysiological field recordings, values for fEPSPs and fiber volleys for slices (n = 7 for control, n = 6 for *Ndufs4(KO)*) were averaged and significance analyzed by one-way ANOVA using GraphPad Prism®. Significance was defined as a p<0.01.

## Supporting information

S1 Fig Loss of NDUFS4 in the PAC. Unmerged and merged confocal images of the (A) Δ–Cre injected and (B) WT-Cre injected PAC (Magnification X1000). White arrows point to cells which retained the red NDUFS4 fluorescence in the absence of virus infection. Scale bar: 10μm.
(TIF)

S2 Fig Cytochrome C immunostaining in the virus injected regions. Unmerged and merged confocal images of the (A) Δ–Cre injected and (B) WT-Cre injected VN (Magnification X1000).
(TIF)

S3 Fig Fluorescent images of brain slices from mice injected with inactive Δ-Cre virus into the (A) VN, (B) MPTA, (C) CMT, (D) DMT and (E) PAC (Magnification X40). Scale bar: 1mm.
(TIF)

S4 Fig Scatter plots showing individual EC_50_ values using ISO and HAL for LORR and TC in the vestibular nucleus (VN), mesopontine tegmental anesthetic area (MPTA), central medial thalamus (CMT), dorsal medial thalamus (DMT) and parietal association cortex (PAC). Large cross bars represent the mean of EC_50_s for ISO (red dots) and HAL (black dots) for the WT-Cre (KD) and Δ-Cre (Control) virus-injected mice in the LORR and TC assays. Small crossbars represent the standard error of the mean. Plots depict viral injections performed into the VN (**A** & **B**, Control n = 6, KD n = 7), MPTA (**C** & **D**, Control n = 7, KD n = 6), CMT (**E** & **F**, n = 6 for Control and KD), DMT (**G** & **H**, n = 6 for Control and KD) and PAC **(I** & **J**, n = 6 for Control and KD).
(TIF)

S1 Table EC_50_s for ISO and HAL for the the global KO and control mice in the LORR assay. Previously published data for the TC assay [3] are included for comparison.
(DOC)

S2 Table Viral injection coordinates and quantities. (DOC)
