# Supplementary figures and images for: Regional knockdown of NDUFS4 implicates a thalamocortical circuit mediating anesthetic sensitivity
Source: PLoS One. 2017 Nov 14;12(11):e0188087. doi: 10.1371/journal.pone.0188087 (PMC5685608; doi:10.1371/journal.pone.0188087)

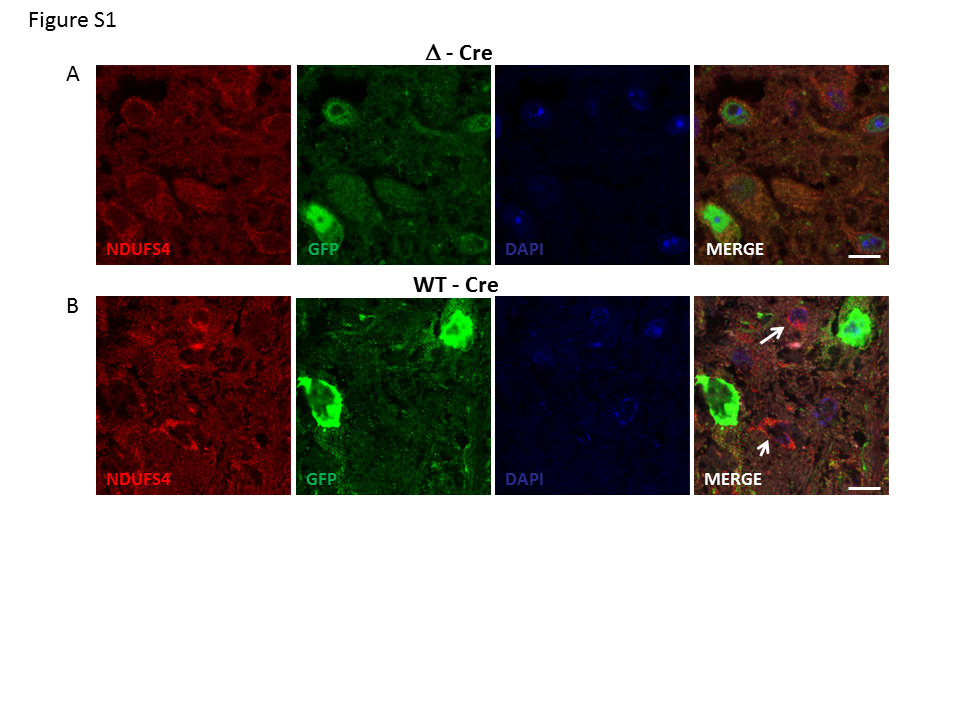

Supplement: S1 Fig — Unmerged and merged confocal images of the (A) Δ–Cre injected and (B) WT-Cre injected PAC (Magnification X1000). White arrows point to cells which retained the red NDUFS4 fluorescence in the absence of virus infection. Scale bar: 10μm. (TIF) [file pone.0188087.s001.TIF]

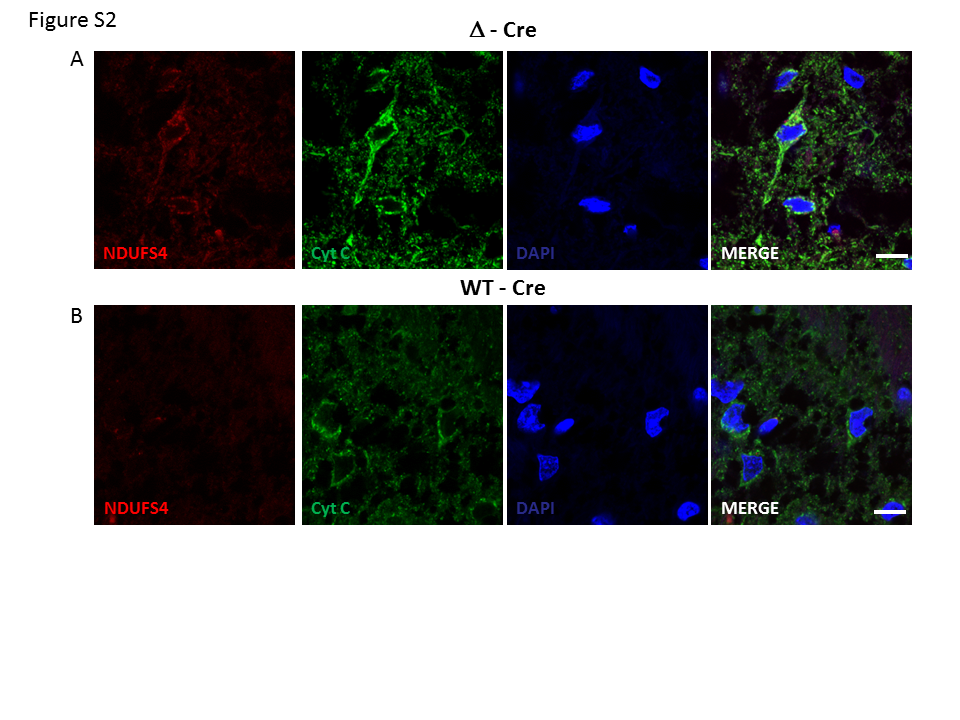

Supplement: S2 Fig — Unmerged and merged confocal images of the (A) Δ–Cre injected and (B) WT-Cre injected VN (Magnification X1000). (TIF) [file pone.0188087.s002.TIF]

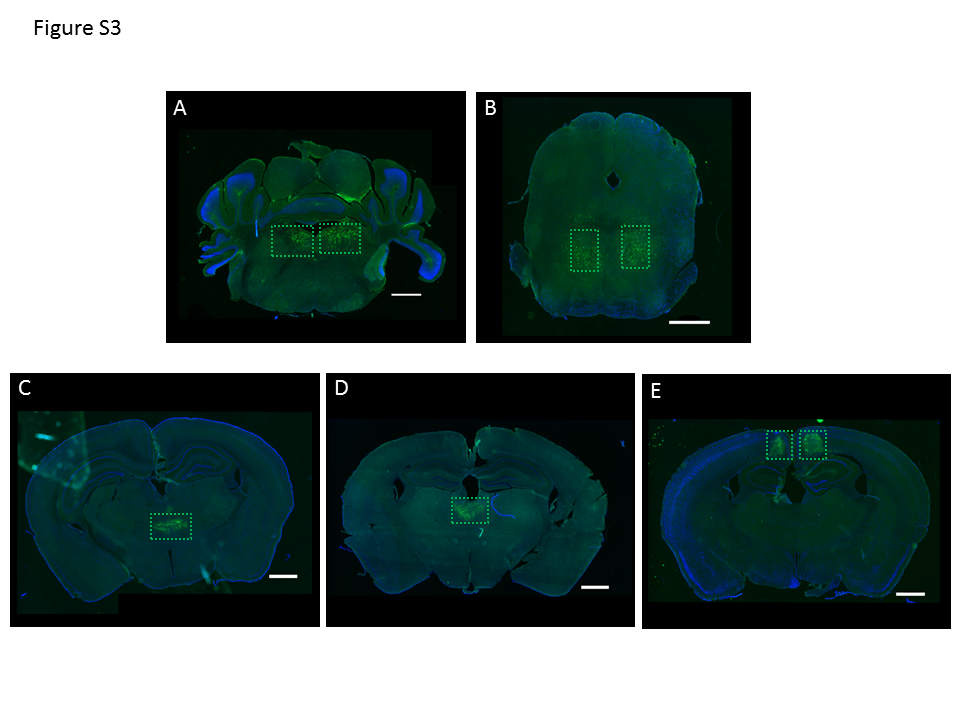

Supplement: S3 Fig — Fluorescent images of brain slices from mice injected with inactive Δ-Cre virus into the (A) VN, (B) MPTA, (C) CMT, (D) DMT and (E) PAC (Magnification X40). Scale bar: 1mm. (TIF) [file pone.0188087.s003.TIF]

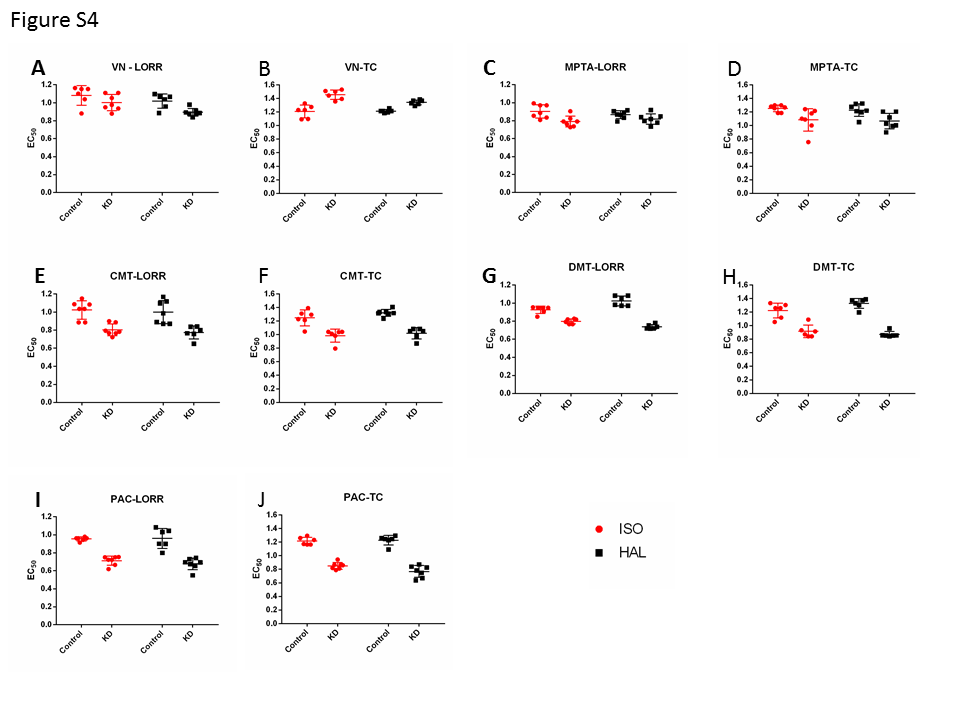

Supplement: S4 Fig — Large cross bars represent the mean of EC50s for ISO (red dots) and HAL (black dots) for the WT-Cre (KD) and Δ-Cre (Control) virus-injected mice in the LORR and TC assays. Small crossbars represent the standard error of the mean. Plots depict viral injections performed into the VN (A & B, Control n = 6, KD n = 7), MPTA (C & D, Control n = 7, KD n = 6), CMT (E & F, n = 6 for Control and KD), DMT (G & H, n = 6 for Control and KD) and PAC (I & J, n = 6 for Control and KD). (TIF) [file pone.0188087.s004.TIF]
