# Supplementary material for: Regional knockdown of NDUFS4 implicates a thalamocortical circuit mediating anesthetic sensitivity
Source: PLoS One. 2017 Nov 14;12(11):e0188087. doi: 10.1371/journal.pone.0188087 (PMC5685608; doi:10.1371/journal.pone.0188087)
Supplement: S1 Table — Previously published data for the TC assay [3] are included for comparison. (DOC) [file pone.0188087.s005.doc]

**Table S1: EC_50_s for ISO and HAL for the the global KO and control mice in the LORR assay.** Previously published data for the TC assay [3] are included for comparison.

|  | | | | | |
| --- | --- | --- | --- | --- | --- |
| **Mouse** | **Genotype** | **Behavioral test** | **EC_50_ ISO (SD)** | **EC_50_ HAL (SD)** | **N** |
| *Ndufs4(KO)* | *Ndufs4* Δ/Δ | LORR | 0.42 (0.08) * | 0.41 (0.05) * | 6 |
| *Ndufs4* het | *Ndufs4* Δ/+ | LORR | 0.96 (0.08) | 0.95 (0.03) | 6 |
| *Ndufs4(KO)* | *Ndufs4* Δ/Δ | TC | 0.44 (0.07) | 0.52 (0.11) | 6 [3] |
| WT | *Ndufs4* +/+ | TC | 1.23 (0.13) | 1.28 (0.07) | 10 [3] |
