# Supplementary material for: Regional knockdown of NDUFS4 implicates a thalamocortical circuit mediating anesthetic sensitivity
Source: PLoS One. 2017 Nov 14;12(11):e0188087. doi: 10.1371/journal.pone.0188087 (PMC5685608; doi:10.1371/journal.pone.0188087)
Supplement: S2 Table — (DOC) [file pone.0188087.s006.doc]

**Table S2:** Viral injection coordinates and quantities

| **Region** | **Co-ordinates from bregma** | **Amount injected** |
| --- | --- | --- |
| Vestibular Nucleus (VN) | ML = ± 1.25; AP = -5.8; DV = 4.3 | 1 ml - bilateral |
| Mesopontine Tegmental Area (MPTA) | ML = ± 0.70; AP = -4.6; DV = 4.1 | 0.6 ml - bilateral |
| Central Medial Thalamus (CMT) | ML = ± 0.32; AP = -1.2; DV = 3.85 | 0.6 ml - bilateral |
| Dorsal Medial Thalamus (DMT) | ML = ± 0.32; AP = -1.2; DV = 3.7 | 0.6 ml - bilateral |
| Parietal Association Cortex (PAC) | ML = ± 0.80; AP = -1.75; DV = 0.9 | 0.6 ml – bilateral |
